# Association between the Fibrosis-4 index and stroke and related outcomes: a meta-analysis

**DOI:** 10.3389/fneur.2026.1791832

**Published:** 2026-08-10

**Authors:** Wei Lai, Haojie Yu, Hongming Wang

**Affiliations:** 1 Graduate School of Zhejiang Chinese Medical University, Hangzhou, Zhejiang, China; 2 The Second School of Clinical Medicine, Zhejiang Chinese Medical University, Hangzhou, Zhejiang, China

**Keywords:** all-cause mortality, Fibrosis-4 index, ischemic stroke, prognosis, stroke

## Abstract

**Objective:**

This meta-analysis evaluated the associations between the Fibrosis-4 index (FIB-4) and overall stroke, ischemic stroke, hemorrhagic outcomes, poor functional outcome, and all-cause mortality.

**Methods:**

PubMed, Embase, and the Cochrane Library were searched from inception to November 4, 2025. Cohort and cross-sectional studies evaluating categorical or continuous FIB-4 were eligible. Quantitative syntheses were stratified by outcome, FIB-4 modeling approach, and effect measure, with odds ratios (ORs) and hazard ratios (HRs) analyzed separately. Prediction intervals were calculated for random-effects analyses containing at least three independent estimates.

**Results:**

Twenty-two studies, including 20 cohort studies and two cross-sectional studies, were included, of which 20 contributed to at least one quantitative synthesis. Elevated categorical FIB-4 was associated with greater odds of overall stroke (OR = 1.86, 95% CI: 1.63–2.14), ischemic stroke (OR = 2.03, 95% CI: 1.72–2.40), symptomatic intracranial hemorrhage (OR = 2.52, 95% CI: 1.79–3.53), poor functional outcome (OR = 2.88, 95% CI: 2.47–3.35), and all-cause mortality (OR = 2.98, 95% CI: 2.51–3.53). Continuous FIB-4 was also associated with overall stroke (HR = 1.08, 95% CI: 1.02–1.14), symptomatic intracranial hemorrhage (OR = 1.33, 95% CI: 1.20–1.48), poor functional outcome (OR = 1.26, 95% CI: 1.11–1.43), and all-cause mortality (HR = 1.08, 95% CI: 1.03–1.12). However, prediction intervals crossed the null for the continuous analyses of poor functional outcome and all-cause mortality, and the categorical overall-stroke analysis was exploratory.

**Conclusion:**

Higher FIB-4 was associated with stroke and adverse stroke-related outcomes across several observational analyses. FIB-4 may provide adjunctive risk information, but prospective validation and formal assessment of its incremental clinical value are required before routine implementation.

**Systematic review registration:**

https://www.crd.york.ac.uk/prospero/search, identifier: CRD420251207801.

## 1 Introduction

Stroke remains a leading cause of death and long-term disability worldwide. In 2021, approximately 93.8 million people were living with stroke, and more than 11 million new cases occurred globally (1). Conventional vascular risk factors account for much of the burden of stroke, but not all of it. Metabolic dysfunction, systemic inflammation, and disturbances in coagulation also contribute to stroke occurrence and recovery. Biomarkers reflecting these processes may offer additional insight into why individuals with otherwise similar clinical profiles experience different outcomes.

Liver fibrosis has been linked to cardiovascular and cerebrovascular disease (2, 3). FIB-4, calculated from age, aspartate aminotransferase, alanine aminotransferase, and platelet count, is widely used as a noninvasive marker in the initial assessment of liver fibrosis (4, 5). Because it relies on routinely collected clinical data, the index can be obtained without additional testing. It may also reflect broader age-related, metabolic, inflammatory, and hematological changes relevant to stroke, although this makes it less specific as a marker of liver fibrosis alone.

Evidence on FIB-4 and stroke has grown, but remains fragmented. Individual studies have examined incident stroke, ischemic stroke, hemorrhagic events, post-treatment sICH, functional outcome, or mortality, often in markedly different populations (3, 6–9). FIB-4 has also been handled differently across studies: some investigators analyzed it continuously, whereas others applied thresholds of 1.30, 1.45, 2.67, or 3.25. These cutoffs were developed for liver-disease assessment rather than for stroke and may not represent comparable exposure across age groups or clinical settings.

Previous systematic reviews have mainly focused on broad cardiovascular outcomes, with stroke often embedded within composite endpoints (10, 11). Evidence specific to stroke and its related outcomes therefore remains uncertain. Findings for ischemic stroke are particularly inconsistent (12), and comparisons across studies are complicated by differences in design, population, outcome definition, FIB-4 coding, follow-up duration, and covariate adjustment (13). ORs and HRs have also sometimes been discussed together, although they represent different measures of association. The same concern applies to categorical and continuous FIB-4 estimates, which address related but distinct questions.

We therefore conducted a systematic review and meta-analysis to examine the associations of FIB-4 with overall stroke, ischemic stroke, hemorrhagic outcomes, poor functional outcome, and all-cause mortality. Categorical and continuous FIB-4 estimates were evaluated separately, as were ORs and HRs. We also examined cutoff-related differences, between-study heterogeneity, and the robustness of the main findings.

## 2 Methods

This study was prospectively registered in the International Prospective Register of Systematic Reviews (PROSPERO; registration number: CRD420251207801), which provided a structured framework for the conduct of the review. The review was conducted and reported in accordance with the PRISMA 2020 statement (14).

### 2.1 Search strategy

Two investigators independently searched PubMed, Embase, and Cochrane Library (inception–November 2025) for eligible cohort and cross-sectional studies. No language restrictions were applied. The search strategy combined Medical Subject Headings (MeSH) terms and free-text keywords, including “Liver Cirrhosis,” “Liver Fibrosis,” “FIB-4,” “Fibrosis-4,” “Stroke,” “cerebrovascular accident,” and “Risk,” and was expanded to cover stroke subtypes (e.g., “ischemic stroke,” “hemorrhagic stroke”) and their related variants. Search strategies were adapted to the specific syntax and indexing systems of each database.

The reference lists of included studies and relevant systematic reviews or meta-analyses were manually screened to identify additional eligible reports. Conference abstracts, trial registrations, and other records without an accessible full original report were not included in the quantitative synthesis. The detailed search strategies are provided in Supplementary Table 1.

### 2.2 Eligibility criteria

Studies were included if they met the following criteria: (1) included adults aged ≥18 years from general-population, cardiovascular/metabolic, or stroke-related clinical cohorts; (2) were original cohort or cross-sectional studies; (3) evaluated FIB-4 as a categorical and/or continuous exposure; and (4) reported an association between FIB-4 and at least one prespecified outcome using an odds ratio (OR), hazard ratio (HR), risk ratio (RR), subdistribution HR, incidence rate ratio, or sufficient group-specific data to calculate an OR, together with a 95% confidence interval (CI).

The primary outcome was overall stroke, defined as a study-reported stroke outcome for which no specific pathological subtype was stipulated or separately restricted. Overall stroke was not constructed by combining separately extracted ischemic and hemorrhagic stroke estimates. Secondary outcomes were ischemic stroke, hemorrhagic stroke and its subtypes, symptomatic intracranial hemorrhage (sICH) after reperfusion treatment, poor functional outcome (generally modified Rankin Scale score >2, equivalent to ≥3), and all-cause mortality. Incident hemorrhagic stroke was analyzed separately from treatment-related sICH.

The exclusion criteria were as follows: (1) Studies were excluded if they were reviews, systematic reviews, case reports, editorials, conference abstracts without a full original report, or other non-original publications; (2) did not report an eligible FIB-4 exposure or prespecified outcome; (3) lacked an effect estimate with a 95% CI and did not provide sufficient data for calculation; or were duplicate or overlapping reports that would contribute non-independent estimates to the same meta-analytic model.

Studies were not excluded solely because FIB-4 was modeled continuously or because only unadjusted group data were available. Adjustment status and the source of each estimate were recorded and considered in the synthesis and interpretation.

**Figure 1:**
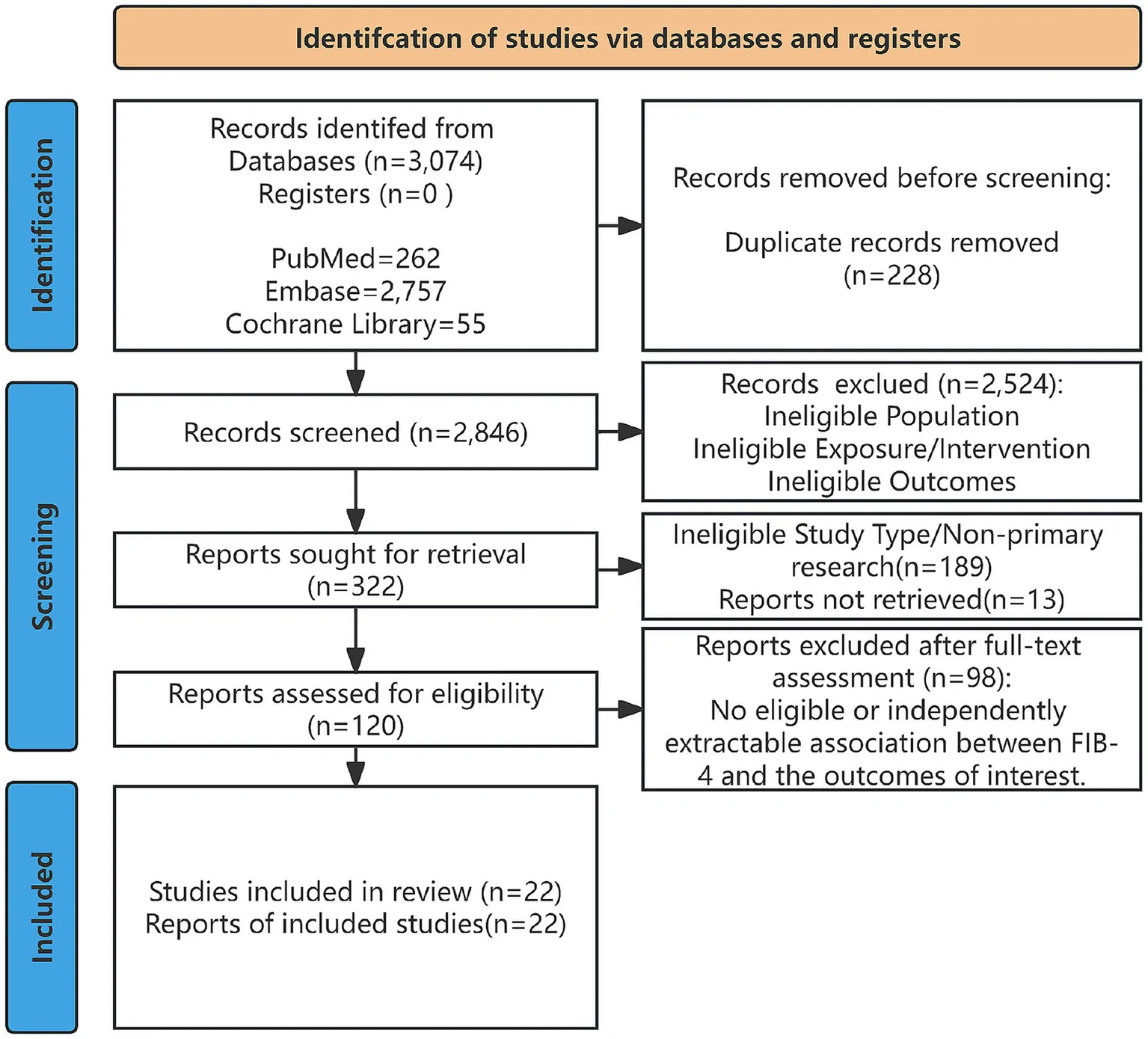
PRISMA flow diagram of study selection.

Two investigators independently screened titles and abstracts and subsequently assessed potentially eligible full texts. Disagreements were resolved through discussion and, when necessary, adjudication by a third investigator. When multiple reports used overlapping cohorts, only one estimate from an independent cohort was included in a given outcome-, exposure-, and effect-measure-specific model. Selection was based on the relevance of the reported outcome and contrast, sample size, completeness of follow-up, and degree of covariate adjustment. Additional eligible estimates from overlapping reports were summarized narratively. The study-selection process followed PRISMA 2020 and is shown in Figure 1.

### 2.3 Study selection and data extraction

Two investigators independently extracted data using a predefined standardized form and cross-checked all entries. Extracted variables included the first author, publication year, country or cohort, study design, sample size, participant characteristics, follow-up duration, FIB-4 modeling approach and cutoff values, outcome definition and assessment time, effect measure, point estimate and 95% CI, numbers of participants and events in each FIB-4 category when available, and covariates included in adjusted models.

When several multivariable models were reported for the same exposure-outcome association, the most fully adjusted compatible estimate was selected. When a study reported multiple outcomes, each eligible outcome was extracted separately. Only one estimate from each independent cohort was included in a given meta-analytic model. If a compatible adjusted binary estimate was unavailable but group-specific sample sizes and event counts were reported, an unadjusted OR and 95% CI were reconstructed. Estimates that could not be harmonized because of incompatible outcome definitions, exposure contrasts, effect measures, or overlapping cohorts were retained for narrative synthesis and are listed in Supplementary Table 7. All extracted and reconstructed values were independently verified by a second investigator.

### 2.4 Quality assessment

Different quality assessment tools were applied according to study design. Cohort studies were evaluated using the Newcastle–Ottawa Scale (NOS), which covers Selection (maximum 4 stars), Comparability (maximum 2 stars), and Outcome (maximum 3 stars). Total NOS scores of 7–9, 5–6, and 0–4 were classified as high, moderate, and low quality, respectively. Cross-sectional studies were evaluated using the 11-item Agency for Healthcare Research and Quality (AHRQ) checklist for cross-sectional/prevalence studies. Each AHRQ item was rated as “Yes,” “No,” or “Unclear”; no numerical total score was calculated. Two investigators performed the assessments independently, with disagreements resolved by discussion or adjudication by a third investigator.

### 2.5 Statistical analysis

All analyses were performed using Stata version 16.0. Meta-analyses were stratified by outcome, FIB-4 modeling approach (categorical or continuous), and effect measure. ORs and HRs were analyzed in separate models on the logarithmic scale and were not treated as interchangeable. Other effect measures were pooled only when at least two independent, clinically comparable estimates of the same measure were available; otherwise, they were summarized narratively. Cohort and cross-sectional evidence was identified explicitly, and cross-sectional estimates were not interpreted as longitudinal risk estimates.

For categorical analyses, FIB-4 was harmonized as a binary exposure. When a study reported a single cutoff, the higher FIB-4 group was compared with the lower group. When two cutoffs were reported, two binary comparisons were reconstructed from group-specific sample sizes and event counts: for the lower cutoff, the intermediate and high groups were combined and compared with the low group; for the upper cutoff, the high group was compared with the combined low and intermediate groups. The upper-cutoff comparison was used in the primary categorical synthesis, and the lower-cutoff comparison was used in cutoff-specific sensitivity analyses. ORs reconstructed from event counts were treated as unadjusted estimates.

Continuous FIB-4 estimates were harmonized to represent the association per 1-unit increase whenever possible. When an estimate was reported per standard-deviation increase, the logarithmic effect estimate and its standard error were divided by the reported standard deviation before exponentiation. Categorical and continuous estimates were never pooled together.

Inverse-variance fixed-effect models were used when statistical heterogeneity was low (I^2^ ≤ 50% and Cochran Q-test *p* > 0.10); otherwise, DerSimonian–Laird random-effects models were used. Heterogeneity was quantified using Cochran’s Q and I^2^ statistics, with Q-test *p* ≤ 0.10 or I^2^ > 50% indicating potentially important heterogeneity. For random-effects analyses containing at least three independent estimates, 95% prediction intervals were calculated to describe the range of effects expected in a future comparable study.

Cutoff-specific sensitivity analyses compared the upper- and lower-cutoff definitions when at least two compatible estimates were available for each cutoff. Leave-one-out sensitivity analyses were performed for principal models containing at least four independent estimates by sequentially omitting one estimate at a time.

For the categorical OR analysis of all-cause mortality, exploratory subgroup analyses were conducted by study design, geographic region, age category, and clinical setting/follow-up duration. *p* values for subgroup differences were obtained from between-subgroup heterogeneity tests. The same moderators were examined separately using univariable random-effects meta-regression with restricted maximum likelihood estimation. Multivariable meta-regression was not performed because only seven independent estimates were available.

Funnel plots and Egger’s regression test were prespecified only for models with at least 10 independent estimates. No quantitative synthesis met this threshold; therefore, formal assessment of small-study effects was not performed. *p* values for pooled effects, heterogeneity, subgroup differences, and meta-regression were reported separately. All tests of pooled effects and moderators were two-sided, with *p* < 0.05 considered statistically significant; *p* ≤ 0.10 was used for Cochran’s Q test.

## 3 Results

### 3.1 Study selection

We systematically searched for studies published before November 4, 2025, and identified 3,074 records (PubMed: 262; Embase: 2,757; Cochrane: 55). After removing 228 duplicates, 2,524 records were excluded based on title/abstract screening for ineligible population, exposure, or outcomes. During the initial eligibility assessment of the remaining 322 articles, 189 non-original studies and 13 studies without accessible original data were excluded. After full-text review, 98 studies were further excluded because they did not report an independent association between FIB-4 and the target outcomes. Ultimately, 22 studies were included in the meta-analysis. The study selection process and results are shown in Figure 1.

### 3.2 Characteristics of the included studies

This meta-analysis included 20 cohort studies and 2 cross-sectional studies published between 2019 and 2025 (6–8, 12, 15–32). Individual study sample sizes ranged from 255 to 452,994 participants. The included studies were conducted across 11 countries and regions, including eight studies based on data from China and four studies based on data from the United States. The mean or median age of participants across studies ranged from 53.2 to 79.5 years. Study populations included community-based adults, cardiovascular or metabolic cohorts, and patients with acute or atrial-fibrillation-related stroke. FIB-4 was modeled categorically, continuously, or both; commonly used thresholds were 1.30, 1.45, 2.67, and 3.25, with age-adjusted thresholds in some studies. Outcomes included overall stroke, ischemic stroke, hemorrhagic stroke and its subtypes, symptomatic intracranial hemorrhage (sICH), poor functional outcome, and all-cause mortality. The main characteristics of the included studies are summarized in Table 1.

**Table 1:** Characteristics of the included studies.

Study	Country/Cohort	Design	*N*	Population	Age, years	Male, %	FIB-4 definition	Outcome	Effect measure	Adjustment	Adjusted covariates	Quality
Xiong et al. (16)	China	Prospective cohort	9,088	Stroke-free rural adults	53.2 ± 10.3	46.1	Continuous; 1.30/2.67	Stroke	HR	Adjusted	Age; sex; drinking; smoking; hypertension; diabetes; LDL-C; total cholesterol; triglycerides; mean waist circumference.	NOS 9/9
Wang et al. (6)	UK Biobank, UK	Prospective cohort	379,953	Adults without prior stroke/CHD	56.2 ± 8.1	44.6	2.67	Stroke; Ischemic stroke; All-cause mortality	HR	Adjusted	age; sex; race; Townsend deprivation index; smoking; alcohol intake; physical activity; diet; hypertension; diabetes; CKD; antihypertensive and lipid-lowering medication use; BMI; waist–hip ratio; cholesterol; CRP	NOS 8/9
Parikh et al. (7)	UK Biobank, UK	Prospective cohort	452,994	Adults aged 40–69 years without hemorrhagic stroke at baseline	56.5 ± 8.1	46.0	2.67	Stroke	HR	Adjusted	age; sex; ethnicity; hypertension; diabetes; dyslipidemia; body mass index; smoking; alcohol consumption frequency; antithrombotic medication use	NOS 9/9
Huang et al. (15)	UKBB/NHANES	Prospective cohort	120,639; 12,907	Hypertensive adults	58.7 ± 7.4; 63.5 ± 12.0	45.6; 46.0	1.30/2.67	Stroke; Ischemic stroke; All-cause mortality	OR	Unadjusted*	Event-count ORs: none.	NOS 8/9
Zhu et al. (31)	China	Prospective cohort	1,135	AIS after IVT	63 (54–70)	72.2	Continuous	Poor functional outcome	OR	Adjusted	age; hypertension; baseline SBP; baseline NIHSS; stroke subtype.	NOS 8/9
Caldonazo et al. (17)	ISCHEMIA trial, US	Post hoc cohort	3,735	CAD trial participants	64 (57–70)	77.6	Continuous	Stroke; All-cause mortality	HR	Adjusted	sex; prior MI; smoking status; diabetes; LVEF <45%; prior CABG; prior heart failure hospitalization; chronic lung disease; prior stroke; known peripheral vascular disease; race; eGFR <60 vs. ≥ 60 mL/min.	NOS 7/9
Toh et al. (24)	Singapore	Retrospective cohort	887	AIS after IVT	67 (57–77)	59.5	1.30/2.67	sICH; Poor functional outcome; All-cause mortality	OR	Adjusted/Unadjusted*	age; admitting NIHSS; BMI; gender; hypertension; hyperlipidemia; diabetes; atrial fibrillation; large-vessel occlusion; TOAST subtype.	NOS 9/9
Parikh et al. (21)	REGARDS, USA	Prospective case-cohort	844	Adults ≥45 years	67.1 ± 11.7	~43	1.45/3.25	Ischemic stroke; Stroke	HR	Adjusted	age; sex; race; Framingham stroke risk factors (hypertension, SBP, diabetes, current smoking, atrial fibrillation, LVH, baseline cardiovascular disease); aspirin; warfarin; age×race interaction.	NOS 8/9
Gao et al. (23)	China	Retrospective cohort	421	LVO-AIS after MT	68 (57–76)	67.2	Continuous; 1.30/2.67	Poor functional outcome; sICH	OR	Adjusted/Unadjusted*	age; baseline NIHSS; baseline GCS; hyperlipidemia; atrial fibrillation; thrombectomy attempts; successful reperfusion; WBC; neutrophils; lymphocytes; platelets; fasting glucose; creatinine; uric acid.	NOS 7/9
Cordero et al. (32)	Spain	Retrospective cohort	3,106	Patients hospitalized for acute coronary syndrome	68.45 ± 12.83	74.25	Continuous	All-cause mortality	OR	Adjusted	age, sex, diabetes, previous coronary heart disease, hemoglobin, revascularization, and GRACE score	NOS 9/9
Fandler-Höfler et al. (28)	Austria	Retrospective cohort	460	Anterior LVO stroke after MT	69.0 ± 13.4	50.7	2.67	Poor functional outcome; All-cause mortality	OR	Adjusted	arterial hypertension; chronic heart disease; diabetes; atrial fibrillation; BMI; alcohol abuse; admission NIHSS; occlusion site; successful recanalization.	NOS 8/9
Norata et al. (25)	Italy	Prospective cohort	264	AIS after IVT	69.3 ± 13.8	53.4	Continuous; 1.30/2.67	Poor functional outcome; sICH	OR	Adjusted/Unadjusted*	Poor-outcome model: female sex; atrial fibrillation; admission NIHSS; EVT; hemoglobin; ANC. sICH model: admission NIHSS, EVT, glucose and ANC.	NOS 9/9
Chen et al. (27)	China	Retrospective cohort	826	AIS after IVT	70 (59–78)	65.0	Continuous	sICH	OR	Adjusted	Age; gender; atrial fibrillation; SBP; TOAST classification; baseline NIHSS; WBC; glucose.	NOS 9/9
Xu et al. (26)	China	Retrospective cohort	578	Anterior LVO-AIS after MT	70.8	58.5	Continuous	sICH	OR	Adjusted	Age, sex, baseline ASPECTS, poor collateral status, baseline NIHSS, successful reperfusion, stroke subtypes, fasting blood glucose	NOS 9/9
Raposeiras-Roubín et al. (22)	Spain	Population cohort	12,870	Atrial fibrillation patients	71.9 ± 10.5	53.8	Continuous; 1.30/2.67	Ischemic stroke; Stroke; All-cause mortality	HR	Adjusted	Age; sex; arterial hypertension; diabetes; prior stroke; coronary artery disease; prior heart failure; COPD; eGFR <60; anemia; LVEF ≤40%; AF type; oral anticoagulation; beta-blockers; renin–angiotensin system inhibitors; statins.	NOS 9/9
Yang et al. (29)	China	Retrospective cohort	522	Cardioembolic stroke with NVAF	72.61 ± 9.77	51.9	Continuous; 1.45/3.25	Poor functional outcome; All-cause mortality	OR/HR	Adjusted/Unadjusted*	Age; BMI; smoking; log NT-proBNP; eGFR; diabetes; hypertension; ischemic heart disease; prior ischemic stroke/TIA.	NOS 7/9
Eto et al. (30)	Japan	Retrospective cohort	1,549	AIS within 7 days	73 ± 12	62.8	Continuous; 1.30/2.67	Poor functional outcome	OR	Adjusted/Unadjusted*	Sex; BMI; daily alcohol intake; hypertension; dyslipidemia; diabetes; CKD; atrial fibrillation; previous stroke; previous ischemic heart disease; NIHSS; hemoglobin; ALP; ChE; albumin; total cholesterol; CRP.	NOS 8/9
Kim et al. (20)	South Korea	Prospective cohort	2,897	AF-related ischemic stroke	75 (68–80)	51.8	Continuous; 3.25	Ischemic stroke; Stroke; All-cause mortality	HR	Adjusted	Sex, congestive heart failure, age, hypertension, diabetes, stroke, vascular disease	NOS 7/9
Song et al. (12)	China	Retrospective cohort	6,563	ACS after PCI	By FIB-4 strata:<1.45, 55.8 ± 9.7; 1.45–3.25, 64.4 ± 9.0; ≥3.25, 65.2 ± 10.5	75.9	1.45/3.25	Ischemic stroke; Stroke; All-cause mortality	OR	Unadjusted*	Event-count ORs: none.	NOS 8/9
Çadırci et al. (8)	Türkiye	Retrospective cohort	255	AIS after IVT	By FIB-4 strata:≤2.67: 67.71 ± 12.54; >2.67: 79.50 ± 9.21	52.9	2.67	Poor functional outcome; sICH; All-cause mortality	OR/HR	Adjusted/Unadjusted*	Age; hypertension; diabetes; atrial fibrillation; admission NIHSS; THRIVE score; 3-month mRS; 3-month mortality.	NOS 6/9
Sun et al. (18)	China	Cross-sectional	1,466	Hospitalized T2DM patients	By groups: SLD-NLF: 47 (38.5, 57); SLD-LF: 62 (52.5, 67.5).	62.6	1.30	Stroke	OR	Adjusted	Drinking; smoking; sex; BMI; diabetes duration; HbA1c; triglycerides.	AHRQ
Parikh et al. (19)	NHANES, USA	Cross-sectional	27,040	Adults without competing liver disease	with NAFLD-fibrosis: 65.5 ± 0.5; without NAFLD-fibrosis: 45.9 ± 0.3	NAFLD-fibrosis group: 49.2%	3.25	Stroke	OR	Adjusted	Age; sex; race/ethnicity; insurance status; poverty; education; physical inactivity; hypertension; diabetes; dyslipidemia; smoking; obesity.	AHRQ

AIS, acute ischemic stroke; CAD, coronary artery disease; CHD, coronary heart disease; HT, hemorrhagic transformation; ICH, intracerebral hemorrhage; IS, ischemic stroke; IVT, intravenous thrombolysis; MACE, major adverse cardiovascular event; MI, myocardial infarction; MT, mechanical thrombectomy; NVAF, nonvalvular atrial fibrillation; OR, odds ratio; HR, hazard ratio; sICH, symptomatic intracranial hemorrhage; TIA, transient ischemic attack. Adjusted = original multivariable estimate; Unadjusted* = event-count OR; Adjusted/Unadjusted* = study contributed both adjusted estimates and event-count ORs or different outcomes were handled differently. Cohort studies were evaluated with NOS; cross-sectional studies were evaluated with AHRQ.

### 3.3 Quality assessment

The mean NOS score across the 20 cohort reports was 8.1 of 9; 19 were rated high quality and 1 moderate quality (Supplementary Table 2). The two cross-sectional studies were assessed using the Cross-Sectional/Prevalence Study Quality Assessment tool. Although some methodological details were incompletely reported, both studies applied multivariable adjustment to control for potential confounding factors, and the overall credibility of their results was considered acceptable (18, 19) (Supplementary Table 3).

### 3.4 Association between FIB-4 and stroke-related outcomes

Eleven quantitative syntheses were conducted across the prespecified outcomes, FIB-4 modeling approaches, and effect measures (Table 2).

**Table 2:** Summary of quantitative syntheses and prediction intervals according to FIB-4 modeling and effect measure.

Outcome	FIB-4 modeling	FIB-4 Coding/Contrast	Effect measure	k	Model	Pooled effect (95% CI)	I^2^ (%)	P for heterogeneity	95% prediction interval
Overall stroke^a^	Categorical	Dichotomized: high vs. lower/non-high FIB-4 group	OR	3	Fixed	1.86 (1.63–2.14)	0.0	0.580	Not applicable
Overall stroke	Continuous	Per 1-unit increase	HR	2	Fixed	1.08 (1.02–1.14)	0.0	0.940	Not applicable
Ischemic stroke	Categorical	Dichotomized: high vs. lower/non-high FIB-4 group	OR	2	Fixed	2.03 (1.72–2.40)	0.0	0.786	Not applicable
Ischemic stroke	Categorical	Dichotomized: high vs. lower/non-high FIB-4 group	HR	3	Random	1.55 (1.01–2.35)	68.3	0.043	0.02–154.21^b^
sICH	Categorical	Dichotomized: high vs. lower/non-high FIB-4 group	OR	4	Fixed	2.52 (1.79–3.53)	0.0	0.486	Not applicable
sICH	Continuous	Per 1-unit increase	OR	2	Fixed	1.33 (1.20–1.48)	0.0	0.707	Not applicable
Poor functional outcome	Categorical	Dichotomized: high vs. lower/non-high FIB-4 group	OR	7	Fixed	2.88 (2.47–3.35)	0.0	0.763	Not applicable
Poor functional outcome	Continuous	Per 1-unit increase	OR	5	Random	1.26 (1.11–1.43)	60.1	0.040	0.86–1.86
All-cause mortality	Categorical	Dichotomized: high vs. lower/non-high FIB-4 group	OR	7	Random	2.98 (2.51–3.53)	51.5	0.054	1.97–4.49
All-cause mortality^c^	Categorical	Dichotomized: high vs. lower/non-high FIB-4 group	HR	2	Random	1.93 (1.07–3.48)	97.3	<0.001	Not estimated (k < 3)
All-cause mortality	Continuous	Per 1-unit increase	HR	4	Random	1.08 (1.03–1.12)	69.6	0.020	0.92–1.26

CI, confidence interval; HR, hazard ratio; OR, odds ratio; PI, prediction interval; sICH, symptomatic intracranial hemorrhage.

Categorical coding: FIB-4 was analyzed as a binary variable. With one cutoff, the high group was compared with the low group. With dual cutoffs (1.30 and 2.67), ORs were reconstructed from event counts as ≥1.30 vs <1.30 and >2.67 vs ≤2.67; the upper-cutoff estimate was used in the primary synthesis and the lower-cutoff estimate in sensitivity analyses.

Models: Fixed-effect inverse-variance models were used for low heterogeneity and DerSimonian-Laird random-effects models otherwise; PIs were estimated for random-effects analyses with at least three independent estimates.

a Exploratory synthesis combining one prospective cohort estimate of incident stroke with two cross-sectional estimates of prevalent stroke.

b Interpret cautiously because only three estimates were available and the PI was extremely wide.

c Interpret cautiously because only two estimates were available and heterogeneity was considerable.

#### 3.4.1 Association between FIB-4 and the risk of overall stroke

**Figure 2:**
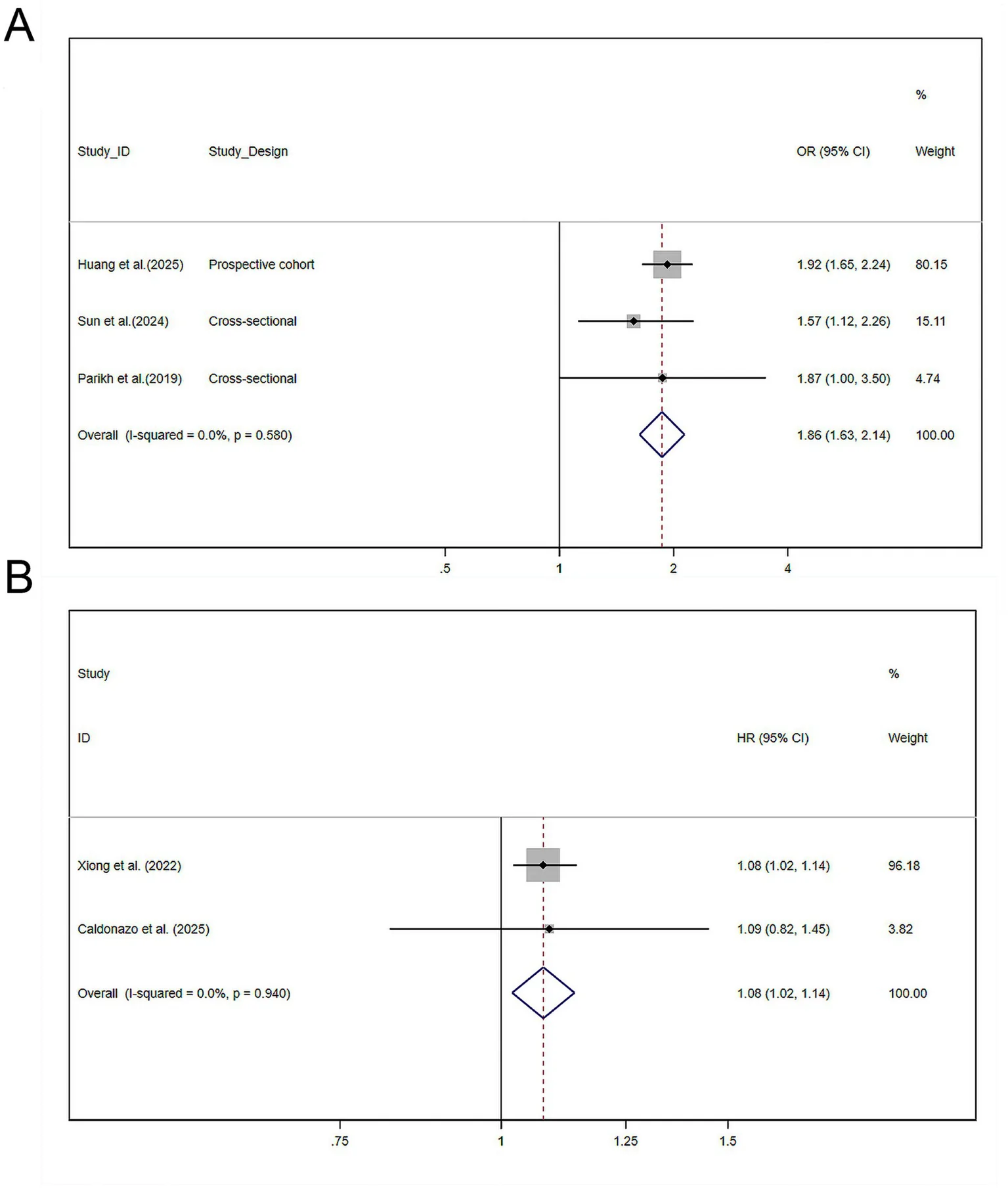
Association between FIB-4 and overall stroke. **(A)** Forest plot of the exploratory OR-based analysis for dichotomized FIB-4 and overall stroke. The pooled OR is exploratory because the model combines one prospective estimate of incident stroke with two cross-sectional estimates of prevalent stroke. **(B)** Forest plot of the HR-based analysis for continuous FIB-4 and overall stroke. HR-based analysis per 1-unit increase.

Three studies contributed to the exploratory categorical analysis (15, 18, 19). Elevated categorical FIB-4 was associated with greater odds of overall stroke (OR = 1.86, 95% CI: 1.63–2.14, *p* < 0.001; I^2^ = 0.0%, P for heterogeneity = 0.580) (Figure 2A). The prospective cohort estimate was OR = 1.93 (95% CI: 1.65–2.24), and the pooled estimate from the two cross-sectional studies was OR = 1.64 (95% CI: 1.21–2.22; I^2^ = 0.0%, P for heterogeneity = 0.633); the between-design difference was not significant (*p* = 0.353). In two cohort studies, each 1-unit increase in FIB-4 was associated with a higher hazard of overall stroke (HR = 1.08, 95% CI: 1.02–1.14, *p* = 0.008; I^2^ = 0.0%, P for heterogeneity = 0.940) (16, 17) (Figure 2B).

#### 3.4.2 Association between FIB-4 and the risk of ischemic stroke

**Figure 3:**
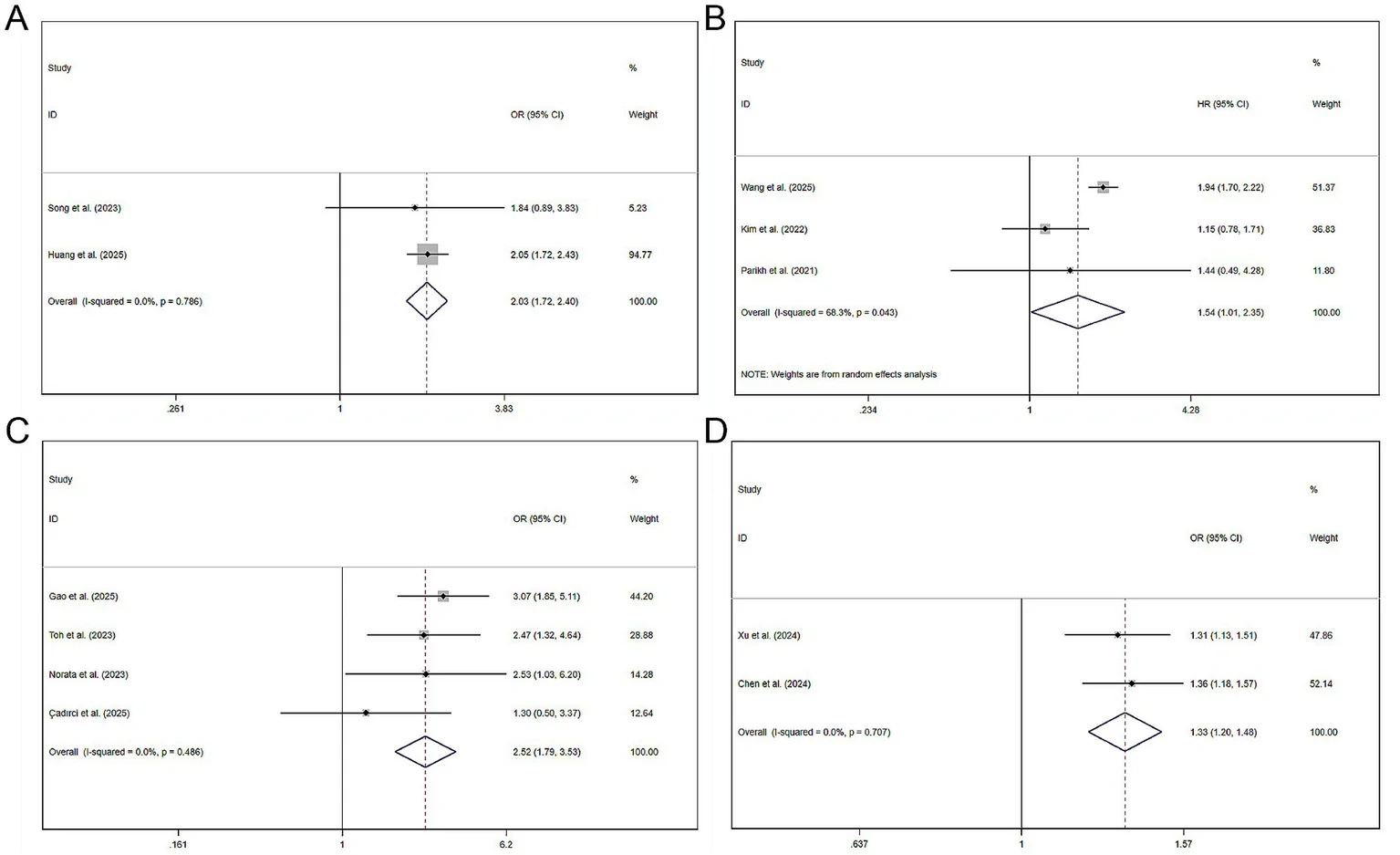
Association between FIB-4 and ischemic stroke and symptomatic intracranial hemorrhage. **(A)** OR-based analysis of dichotomized FIB-4 and ischemic stroke. **(B)** HR-based analysis of dichotomized FIB-4 and ischemic stroke. **(C)** OR-based analysis of dichotomized FIB-4 and symptomatic intracranial hemorrhage. **(D)** OR-based analysis of continuous FIB-4 and symptomatic intracranial hemorrhage.

Two studies contributed categorical ORs (12, 15). Elevated categorical FIB-4 was associated with greater odds of ischemic stroke (OR = 2.03, 95% CI: 1.72–2.40, *p* < 0.001; I^2^ = 0.0%, P for heterogeneity = 0.786) (Figure 3A). Three studies contributed categorical HRs (6, 20, 21). Elevated categorical FIB-4 was associated with a higher hazard of ischemic stroke (HR = 1.55, 95% CI: 1.01–2.35, *p* = 0.043; I^2^ = 68.3%, P for heterogeneity = 0.043; 95% prediction interval, 0.02–154.21) (Figure 3B). One additional study reported no association between continuous FIB-4 and ischemic stroke (HR = 1.00, 95% CI: 0.96–1.05) (22) (Supplementary Table 7).

#### 3.4.3 Association between FIB-4 and hemorrhagic outcomes

Two overlapping UK Biobank analyses reported positive associations between FIB-4 > 2.67 and incident hemorrhagic outcomes (6, 7). The reported HRs were 2.00 (95% CI: 1.60–2.60) for hemorrhagic stroke, 2.00 (95% CI: 1.50–2.70) and 2.14 (95% CI: 1.63–2.81) for intracerebral hemorrhage, and 2.20 (95% CI: 1.50–3.50) and 1.90 (95% CI: 1.27–2.84) for subarachnoid hemorrhage; these estimates were not pooled (Supplementary Table 7).

Four studies contributed categorical ORs for sICH (11, 26–28). Elevated categorical FIB-4 was associated with greater odds of sICH (OR = 2.52, 95% CI: 1.79–3.53, *p* < 0.001; I^2^ = 0.0%, P for heterogeneity = 0.486) (Figure 3C). Two studies contributed continuous ORs (29, 30). Each 1-unit increase in FIB-4 was associated with greater odds of sICH (OR = 1.33, 95% CI: 1.20–1.48, *p* < 0.001; I^2^ = 0.0%, P for heterogeneity = 0.707) (Figure 3D).

#### 3.4.4 Association between FIB-4 and poor functional outcomes

**Figure 4:**
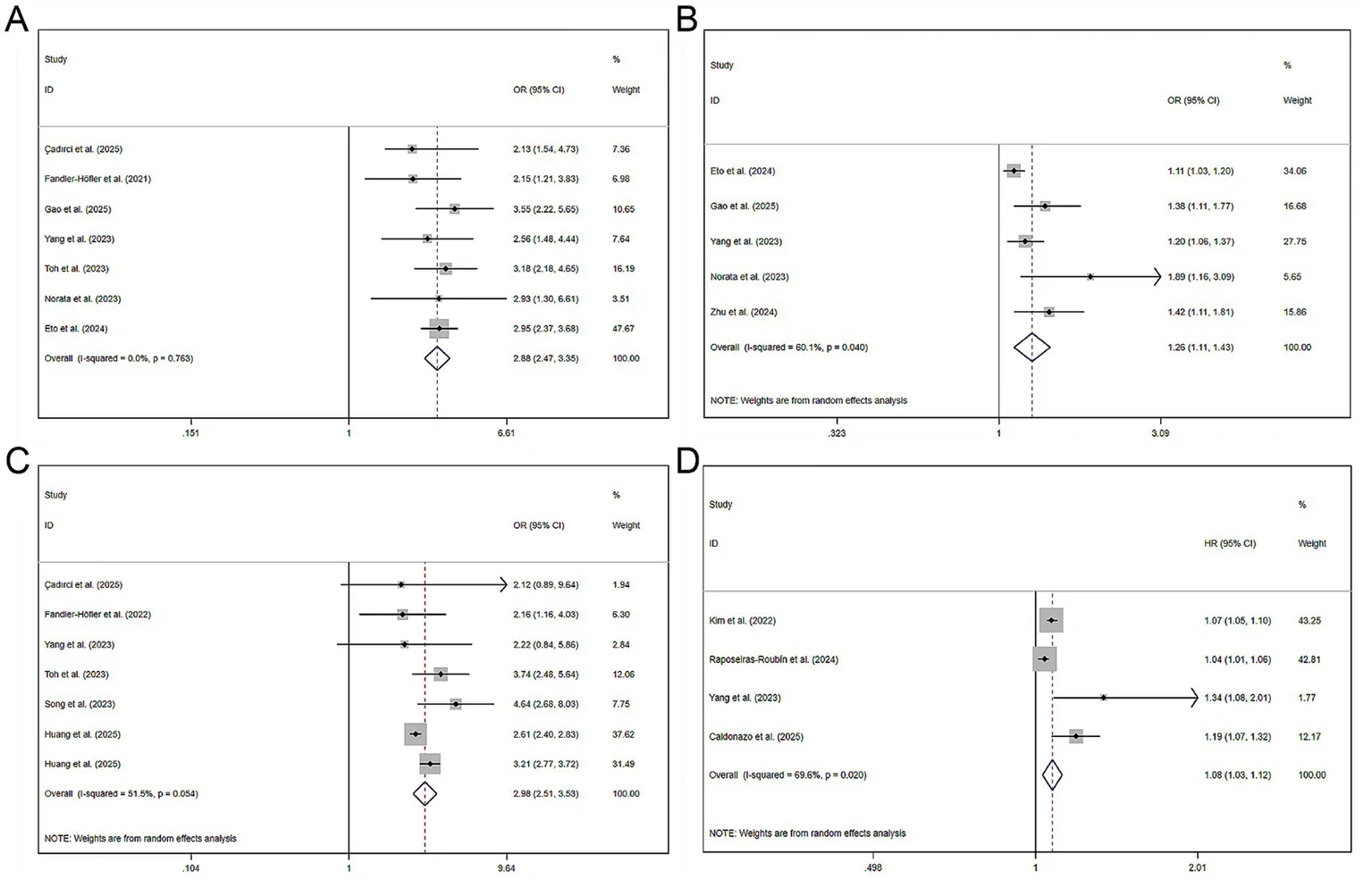
Association between FIB-4 and poor functional outcome and all-cause mortality. **(A)** OR-based analysis of dichotomized FIB-4 and poor functional outcome. **(B)** OR-based analysis of continuous FIB-4 and poor functional outcome. **(C)** OR-based analysis of dichotomized FIB-4 and all-cause mortality. **(D)** HR-based analysis of continuous FIB-4 and all-cause mortality.

Seven studies evaluated categorical FIB-4 in relation to poor functional outcome, generally defined as a modified Rankin Scale score of at least 3 (8, 23–25, 28–30). Elevated categorical FIB-4 was associated with greater odds of poor functional outcome (OR = 2.88, 95% CI: 2.47–3.35, *p* < 0.001; I^2^ = 0.0%, P for heterogeneity = 0.763) (Figure 4A). Five studies evaluated continuous FIB-4 (23, 25, 29–31). Each 1-unit increase in FIB-4 was associated with greater odds of poor functional outcome (OR = 1.26, 95% CI: 1.11–1.43, *p* < 0.001; I^2^ = 60.1%, P for heterogeneity = 0.040; 95% prediction interval, 0.86–1.86) (Figure 4B).

#### 3.4.5 Association between FIB-4 and all-cause mortality

Seven independent cohort estimates contributed to the categorical OR analysis (8, 12, 15, 24, 28, 29). Elevated categorical FIB-4 was associated with greater odds of all-cause mortality (OR = 2.98, 95% CI: 2.51–3.53, p < 0.001; I^2^ = 51.5%, P for heterogeneity = 0.054; 95% prediction interval, 1.97–4.49) (Figure 4C). Two studies contributed categorical HRs (6, 20). Elevated categorical FIB-4 was associated with a higher hazard of all-cause mortality (HR = 1.93, 95% CI: 1.07–3.48, *p* = 0.029; I^2^ = 97.3%, P for heterogeneity < 0.001). Four studies evaluated continuous FIB-4 (17, 20, 22, 29). Each 1-unit increase in FIB-4 was associated with a higher hazard of all-cause mortality (HR = 1.08, 95% CI: 1.03–1.12, *p* = 0.001; I^2^ = 69.6%, P for heterogeneity = 0.020; 95% prediction interval, 0.92–1.26) (Figure 4D). One additional acute coronary syndrome cohort reported associations of continuous FIB-4 (adjusted OR = 1.24, 95% CI: 1.04–1.47) and FIB-4 > 2.67 versus <1.30 (adjusted OR = 2.35, 95% CI: 1.05–5.28) with in-hospital all-cause mortality (32); these estimates were not pooled (Supplementary Table 7).

### 3.5 Cutoff-specific and leave-one-out sensitivity analyses

Cutoff-specific analyses showed positive associations at both lower and upper thresholds, although statistical significance differed for overall stroke. For overall stroke, elevated FIB-4 was significantly associated with greater odds of stroke at the upper cutoff (OR = 1.92, 95% CI: 1.66–2.23; I^2^ = 0.0%), whereas the lower-cutoff analysis was not statistically significant (OR = 1.27, 95% CI: 0.92–1.74; I^2^ = 71.0%; *p* = 0.144). For sICH, the pooled ORs were 2.11 (95% CI: 1.40–3.19; I^2^ = 0.0%) and 2.52 (95% CI: 1.79–3.53; I^2^ = 0.0%) at the lower and upper cutoffs, respectively. The corresponding ORs were 2.48 (95% CI: 2.09–2.95; I^2^ = 0.0%) and 2.88 (95% CI: 2.47–3.35; I^2^ = 0.0%) for poor functional outcome, and 1.86 (95% CI: 1.39–2.47; I^2^ = 94.7%) and 2.98 (95% CI: 2.51–3.53; I^2^ = 51.5%) for all-cause mortality (Supplementary Figure 1).

In leave-one-out analyses, the associations of categorical FIB-4 with sICH, poor functional outcome, and all-cause mortality remained statistically significant after sequential exclusion of individual estimates. The association between continuous FIB-4 and poor functional outcome was also unchanged (Supplementary Figure 2).

### 3.6 Exploratory subgroup analyses for all-cause mortality

Elevated categorical FIB-4 was associated with greater odds of all-cause mortality in all prespecified subgroups. The pooled associations were similar between retrospective and prospective cohorts (OR = 3.24, 95% CI: 2.37–4.42 vs. OR = 2.86, 95% CI: 2.34–3.51; P for subgroup difference = 0.185), between middle-aged and older populations (OR = 3.00, 95% CI: 2.44–3.68 vs. OR = 2.93, 95% CI: 1.99–4.33; P for subgroup difference = 0.562), and between acute-stroke/short-term and cardiovascular-metabolic/long-term studies (OR = 2.97, 95% CI: 2.17–4.06 vs. OR = 3.04, 95% CI: 2.44–3.79; P for subgroup difference = 0.659). The pooled OR was higher in Asian than in Western studies (OR = 3.80, 95% CI: 2.78–5.19 vs. OR = 2.79, 95% CI: 2.36–3.29; P for subgroup difference = 0.042) (Supplementary Table 4).

### 3.7 Univariable meta-regression analyses of all-cause mortality

Univariable meta-regression did not identify significant moderation of the association between categorical FIB-4 and all-cause mortality by study design (ratio of ORs = 0.87, 95% CI: 0.62–1.23; *p* = 0.443), geographic region (ratio of ORs = 0.74, 95% CI: 0.51–1.06; *p* = 0.103), age category (ratio of ORs = 0.99, 95% CI: 0.65–1.51; *p* = 0.960), or clinical setting/follow-up duration (ratio of ORs = 1.06, 95% CI: 0.70–1.61; *p* = 0.789) (Supplementary Table 5).

## 4 Discussion

### 4.1 Interpretation of the main findings

This meta-analysis of 22 observational studies found that higher FIB-4 was associated with overall stroke, ischemic stroke, sICH, poor functional outcome, and all-cause mortality. The clearest and most consistent associations were seen for sICH, poor functional outcome, and mortality in the categorical analyses. Evidence for stroke occurrence was less straightforward. The overall-stroke OR combined one prospective estimate of incident stroke with two cross-sectional estimates of prevalent stroke, while the ischemic-stroke HR analysis showed substantial heterogeneity and an exceptionally wide prediction interval. FIB-4 may therefore capture clinically relevant risk across several stroke-related settings, although the magnitude of the association is unlikely to be uniform and the available data do not support a causal interpretation.

Evidence on stroke occurrence came mainly from prospective cohorts in which higher FIB-4 was associated with incident stroke (6, 16). The two cross-sectional studies added information on prevalent stroke, but could not establish whether elevated FIB-4 preceded the event. The pooled categorical estimate for overall stroke is therefore best viewed as exploratory. In the continuous analysis, stroke hazard increased modestly with each 1-unit increase in FIB-4. A similar association has been reported in patients with acute coronary syndrome (32), although that population differs from the stroke cohorts included here.

Associations with post-stroke outcomes were more consistent in the categorical OR analyses. Higher FIB-4 was associated with sICH, poor functional outcome, and all-cause mortality, in keeping with findings from acute ischemic stroke and large-vessel-occlusion cohorts (28). These estimates should not be compared directly with those for stroke occurrence because the populations, treatments, outcome definitions, and follow-up periods differed. Continuous models produced smaller relative effects, and the prediction intervals for poor functional outcome and mortality included the null. FIB-4 may carry prognostic information across its range, but the effect expected in a new clinical setting remains uncertain.

### 4.2 FIB-4 as a context-dependent risk marker

FIB-4 is better understood as a composite clinical marker than as a direct measure of hepatic fibrosis. Age, aspartate aminotransferase, alanine aminotransferase, and platelet count each reflect more than liver status; together, they may also capture ageing, acute illness, metabolic burden, and hematological change. Two patients with the same score may therefore have quite different underlying conditions. This is particularly relevant in stroke research because age is built into the index and is itself a major determinant of stroke risk and prognosis. Conventional FIB-4 thresholds become less specific with advancing age (33), and statistical adjustment cannot completely remove this structural overlap.

Timing also matters. In population-based cohorts, FIB-4 probably reflects pre-existing hepatic and cardiometabolic risk. During acute stroke, however, aminotransferase levels and platelet counts may change in response to physiological stress, infection, treatment, nutritional status, or stroke severity. A value obtained on admission may therefore reflect both chronic vulnerability and the acute event itself. This makes reverse causation a genuine concern in prognostic studies. Future studies should distinguish pre-stroke, admission, and serial FIB-4 measurements rather than treating them as equivalent.

The observed associations are biologically plausible, although no single pathway is likely to account for them. Metabolic liver disease clusters with obesity, diabetes, hypertension, and dyslipidemia, all of which increase vascular risk, and hepatic fibrosis has been linked to subsequent cardiovascular events (2). Liver disease can also alter both procoagulant and anticoagulant pathways, offering a possible connection to ischemic as well as hemorrhagic complications (34). A further link may be atrial fibrillation, which has been associated with higher FIB-4 in patients with acute ischemic stroke (35). Even so, the present data cannot separate an effect of liver fibrosis itself from the broader cardiometabolic and systemic burden reflected by FIB-4.

### 4.3 Heterogeneity and methodological considerations

Differences in how FIB-4 was defined are an important source of uncertainty. The commonly used thresholds were not developed in stroke populations. In patients with HIV/HCV coinfection, a value below 1.45 had a negative predictive value of 90% for excluding advanced fibrosis, whereas a value above 3.25 had a specificity of 97% for identifying advanced fibrosis (4). In nonalcoholic fatty liver disease, thresholds of 1.30 and 2.67 yielded a negative predictive value of 90% and a positive predictive value of 80%, respectively (36). Some included cohorts also used age-adjusted definitions. These thresholds are not interchangeable, and the same numerical value may not represent the same biological state across populations.

Results from the cutoff analyses mostly pointed in the same direction, although the lower-cutoff estimate for overall stroke was not statistically significant. The larger effects observed at upper thresholds require caution. Raising the cutoff changes both the exposed and reference groups, so the difference cannot be taken as evidence of a dose–response relationship or more severe histological fibrosis. Continuous modeling avoids some of this arbitrariness, but a 1-unit increase in FIB-4 may not have exactly the same meaning across age groups, populations, and clinical settings.

Heterogeneity was uneven across the analyses. Several categorical models had low I^2^ values, but some were based on only two to four estimates, so apparent homogeneity may simply reflect limited power. The categorical HR analysis for ischemic stroke and several continuous or mortality analyses were clearly more variable. Prediction intervals remained above 1 only for categorical FIB-4 and all-cause mortality; they crossed the null for ischemic-stroke HRs, continuous poor functional outcome, and continuous mortality. Neither subgroup analyses nor meta-regression identified a consistent explanation for this variation. The nominal regional difference seen in the subgroup analysis was not reproduced in meta-regression and may have arisen by chance. Leave-one-out analyses showed that the principal estimates were not driven by a single study, but they do not address residual confounding or other systematic bias.

### 4.4 Strengths, limitations, and clinical implications

Several features strengthen this analysis. Categorical and continuous FIB-4 were examined separately, ORs and HRs were not combined, prediction intervals were reported where appropriate, and overlapping cohorts were handled to avoid double counting. The limitations are more important to the interpretation. All included studies were observational, two were cross-sectional, and some ORs were reconstructed from event counts without adjustment. Populations, treatments, outcome definitions, follow-up periods, thresholds, and the timing of FIB-4 measurement also varied. Overlap among UK Biobank and NHANES reports further reduced the effective independence of the evidence and made a unique participant total unreliable.

Many syntheses included only a few estimates. Between-study variance and prediction intervals are therefore imprecise, even when the pooled effect is statistically significant. No model contained 10 or more independent estimates, so funnel plots and Egger tests would not have been informative. High NOS scores are reassuring about study design and reporting, but they do not remove selection bias, exposure misclassification, reverse causation, or residual confounding. The included studies also evaluated association rather than predictive performance. Whether FIB-4 adds useful information beyond age, stroke severity, atrial fibrillation, and established vascular risk factors remains unknown.

FIB-4 is inexpensive and readily available, which makes it an attractive candidate marker. The present results do not support using it alone to guide treatment or prognosis. Prospective studies should prespecify age-appropriate thresholds, standardize the timing of laboratory measurement, and report categorical and continuous estimates with comparable adjustment. Serial measurements may prove more informative than a single value. Individual-participant-data analyses could address non-linearity and interactions with age or treatment, while prediction studies should test whether adding FIB-4 improves discrimination, calibration, and clinical decision-making beyond existing models.

Higher FIB-4 was associated with overall stroke, ischemic stroke, sICH, poor functional outcome, and all-cause mortality, but the evidence was not equally robust across outcomes. FIB-4 appears to be a context-dependent marker of hepatic, metabolic, and systemic risk rather than a direct measure of causal liver fibrosis. Its clinical use will depend on prospective validation and evidence that it adds meaningful information to established risk models.

## Data Availability

The raw data supporting the conclusions of this article will be made available by the authors, without undue reservation.

## Supplementary material

SUPPLEMENTARY FIGURE 1 Cutoff-specific sensitivity analyses of dichotomized FIB-4. **(A)** Overall stroke. **(B)** Symptomatic intracranial hemorrhage. **(C)** Poor functional outcome. **(D)** All-cause mortality.

SUPPLEMENTARY FIGURE 2 Leave-one-out sensitivity analyses. **(A)** Dichotomized FIB-4 and sICH; **(B)** Dichotomized FIB-4 and poor functional outcome; **(C)** Continuous FIB-4 and poor functional outcome; **(D)** Dichotomized FIB-4 and all-cause mortality.
