# Supplementary material for: Association between the Fibrosis-4 index and stroke and related outcomes: a meta-analysis
Source: Front Neurol. 2026 Aug 10;17:1791832. doi: 10.3389/fneur.2026.1791832 (PMC13504023; doi:10.3389/fneur.2026.1791832)
Supplement: Supplementary file 1 [file Table_1.docx]

Supplementary Material

**Supplementary Table 1.** The retrieval strategies and retrieval results of each database are shown in Tables 1-3

Table 1a. PubMed 2025-11-04

| No. | Content | Result |
| --- | --- | --- |
| #1 | Search: "Liver Cirrhosis"[Mesh] Sort by: Most Recent | 110, 112 |
| #2 | Search: (Hepatic Cirrhosis[Title/Abstract]) OR (Liver Fibrosis[Title/Abstract]) Sort by: Most Recent | 31,825 |
| #3 | Search: ("Liver Cirrhosis"[Mesh]) OR ((Hepatic Cirrhosis[Title/Abstract]) OR (Liver Fibrosis[Title/Abstract])) Sort by: Most Recent | [122,407](https://pubmed.ncbi.nlm.nih.gov/?term=() |
| #4 | Search: ((FIB‐4[Title/Abstract]) OR (FIB4[Title/Abstract])) OR (fibrosis‐4[Title/Abstract]) Sort by: Most Recent | 4,513 |
| #5 | Search: "Stroke"[Mesh] Sort by: Most Recent | 195,311 |
| #6 | Search: (((Stroke*[Title/Abstract]) OR (Cerebrovascular[Title/Abstract])) OR (Brain Vascular Accident*[Title/Abstract])) OR (Apoplexy[Title/Abstract]) Sort by: Most Recent | 435,192 |
| #7 | Search: ("Stroke"[Mesh]) OR ((((Stroke*[Title/Abstract]) OR (Cerebrovascular[Title/Abstract])) OR (Brain Vascular Accident*[Title/Abstract])) OR (Apoplexy[Title/Abstract])) Sort by: Most Recent | 472,117 |
| #8 | Search: "Risk"[Mesh] Sort by: Most Recent | 1,502,350 |
| #9 | Search: Risk*[Title/Abstract] Sort by: Most Recent | 3,500,883 |
| #10 | Search: ("Risk"[Mesh]) OR (Risk*[Title/Abstract]) Sort by: Most Recent | 4,008,554 |
| #11 | Search: (((("Liver Cirrhosis"[Mesh]) OR ((Hepatic Cirrhosis[Title/Abstract]) OR (Liver Fibrosis[Title/Abstract]))) OR (((FIB‐4[Title/Abstract]) OR (FIB4[Title/Abstract])) OR (fibrosis‐4[Title/Abstract]))) AND (("Stroke"[Mesh]) OR ((((Stroke*[Title/Abstract]) OR (Cerebrovascular[Title/Abstract])) OR (Brain Vascular Accident*[Title/Abstract])) OR (Apoplexy[Title/Abstract])))) AND (("Risk"[Mesh]) OR (Risk*[Title/Abstract])) Sort by: Most Recent | 262 |

Table 1b. Embase 2025-11-04

| No. | Content | Result |
| --- | --- | --- |
| #1 | 'liver cirrhosis'/exp | 233,288 |
| #2 | 'hepatic cirrhosis':ab,ti OR 'liver fibrosis':ab,ti | 50,529 |
| #3 | #1 OR #2 | 266,326 |
| #4 | 'fib‐4':ab,ti OR 'fib4':ab,ti OR 'fibrosis‐4':ab,ti | 137,405 |
| #5 | 'cerebrovascular accident'/exp | 528,265 |
| #6 | 'stroke*':ab,ti OR 'cerebrovascular':ab,ti OR 'brain vascular accident*':ab,ti OR 'apoplexy':ab,ti | 682,616 |
| #7 | #5 OR #6 | 807,283 |
| #8 | 'risk'/exp | 3,588,714 |
| #9 | 'risk*':ab,ti | 5,096,083 |
| #10 | #8 OR #9 | 6,050,178 |
| #11 | #3 OR #4 | 374,413 |
| #12 | #7 AND #10 AND #11 | 2,757 |

Table 1c. Cochrane Library 2025-11-04

| No. | Content | Result |
| --- | --- | --- |
| #1 | MeSH descriptor: [Liver Cirrhosis] explode all trees | 4159 |
| #2 | (Hepatic Cirrhosis):ab,ti,kw OR (Liver Fibrosis):ab,ti,kw | 8195 |
| #3 | #1 OR #2 | 10164 |
| #4 | (FIB‐4):ab,ti,kw OR (FIB4):ab,ti,kw OR (fibrosis‐4):ab,ti,kw | 590 |
| #5 | MeSH descriptor: [Stroke] explode all trees | 18648 |
| #6 | (Stroke*):ab,ti,kw OR (Cerebrovascular):ab,ti,kw OR (Brain Vascular Accident*):ab,ti,kw OR (Apoplexy):ab,ti,kw | 89339 |
| #7 | #5 OR #6 | 89816 |
| #8 | MeSH descriptor: [Risk] explode all trees | 56200 |
| #9 | (Risk*):ab,ti,kw | 341151 |
| #10 | #8 OR #9 | 344969 |
| #11 | #3 OR #4 | 10281 |
| #12 | #7 AND #10 AND #11 | 55 |

**Supplementary Table 2.** Quality Assessment of Cohort Studies Using the Newcastle–Ottawa Scale (NOS)

| **NOS domain** | **Assessment criterion** | **Parikh et al. (2025)** | **Wang et al. (2025)** | **Gao et al. (2025)** | **Huang et al. (2025)** | **Cordero et al.（2023）** | **Caldonazo et al. (2025)** | **Raposeiras-Roubín et al. (2024)** | **Eto et al. (2024)** | **Xu et al. (2024)** | **Chen et al. (2024)** |
| --- | --- | --- | --- | --- | --- | --- | --- | --- | --- | --- | --- |
| Selection (maximum 4 stars) | (1) Representativeness of the exposed cohort | ★ | ★ | ★ | ★ | ★ | ★ | ★ | ★ | ★ | ★ |
|  | (2) Selection of the non-exposed cohort | ★ | ★ | ★ | ★ | ★ | ★ | ★ | ★ | ★ | ★ |
|  | (3) Ascertainment of exposure | ★ | ★ | ★ | ★ | ★ | ★ | ★ | ★ | ★ | ★ |
|  | (4) Demonstration that the outcome of interest was not present at the start of the study | ★ | ★ | ★ | ★ | ★ | ★ | ★ | ★ | ★ | ★ |
| Comparability (maximum 2 stars) | (1) Comparability based on study design or analysis (most important confounder) | ★ | — | ★ | ★ | ★ | — | ★ | ★ | ★ | ★ |
|  | (2) Control for additional factors | ★ | ★ | ★ | ★ | ★ | ★ | ★ | ★ | ★ | ★ |
| Outcome (maximum 3 stars) | (1) Assessment of outcome | ★ | ★ | — | ★ | ★ | ★ | ★ | — | ★ | ★ |
|  | (2) Follow-up long enough for outcomes to occur | ★ | ★ | ★ | ★ | ★ | ★ | ★ | ★ | ★ | ★ |
|  | (3) Adequacy of follow-up of cohorts | ★ | ★ | — | — | ★ | — | ★ | ★ | ★ | ★ |
| **Total NOS score (maximum 9)** | | **9/9** | **8/9** | **7/9** | **8/9** | **9/9** | **7/9** | **9/9** | **8/9** | **9/9** | **9/9** |

**Supplementary Table 2.** Continued

| **NOS domain** | **Assessment criterion** | **Zhu et al. (2024)** | **Yang et al. (2023)** | **Toh et al. (2023)** | **Norata et al. (2023)** | **Song et al. (2023)** | **Xiong et al. (2022)** | **Kim et al. (2022)** | **Fandler-Höfler et al. (2021)** | **Parikh et al. (2021)** | **Çadırci et al. (2025)** |
| --- | --- | --- | --- | --- | --- | --- | --- | --- | --- | --- | --- |
| Selection (maximum 4 stars) | (1) Representativeness of the exposed cohort | ★ | ★ | ★ | ★ | ★ | ★ | ★ | ★ | ★ | ★ |
|  | (2) Selection of the non-exposed cohort | ★ | ★ | ★ | ★ | ★ | ★ | ★ | ★ | ★ | ★ |
|  | (3) Ascertainment of exposure | ★ | ★ | ★ | ★ | ★ | ★ | ★ | ★ | ★ | ★ |
|  | (4) Demonstration that the outcome of interest was not present at the start of the study | ★ | ★ | ★ | ★ | ★ | ★ | ★ | ★ | ★ | ★ |
| Comparability (maximum 2 stars) | (1) Comparability based on study design or analysis (most important confounder) | ★ | — | ★ | ★ | ★ | ★ | ★ | ★ | ★ | — |
|  | (2) Control for additional factors | ★ | ★ | ★ | ★ | ★ | ★ | ★ | ★ | ★ | — |
| Outcome (maximum 3 stars) | (1) Assessment of outcome | — | — | ★ | ★ | ★ | ★ | — | — | ★ | — |
|  | (2) Follow-up long enough for outcomes to occur | ★ | ★ | ★ | ★ | ★ | ★ | ★ | ★ | ★ | ★ |
|  | (3) Adequacy of follow-up of cohorts | ★ | ★ | ★ | ★ | — | ★ | — | ★ | — | ★ |
| **Total NOS score (maximum 9)** | | **8/9** | **7/9** | **9/9** | **9/9** | **8/9** | **9/9** | **7/9** | **8/9** | **8/9** | **6/9** |

**Note: The cohort version of the NOS assesses Selection (maximum 4 stars), Comparability (maximum 2 stars), and Outcome (maximum 3 stars), for a total of 9 stars. A filled star (★) indicates that the criterion was met; an em dash (—) indicates that it was not met. For this review, total scores of 7–9, 5–6, and 0–4 were classified as high, moderate, and low quality, respectively.**

**Supplementary Table 3.** Quality assessment of cross-sectional studies using the AHRQ checklist

| Item | Sun et al.  (2024) | | | Parikh et al.  (2019) | | |
| --- | --- | --- | --- | --- | --- | --- |
|  | Yes | No | Unclear | Yes | No | Unclear |
| 1) Define the source of information (survey, record review) | ✔ |  |  | ✔ |  |  |
| 2) List inclusion and exclusion criteria for exposed and unexposed subjects | ✔ |  |  | ✔ |  |  |
| 3) Indicate time period used for identifying patients | ✔ |  |  | ✔ |  |  |
| 4) Indicate whether or not subjects were consecutive if not population-based |  |  | ✔ | ✔ |  |  |
| 5) Indicate if evaluators of subjective components were masked |  | ✔ |  |  |  | ✔ |
| 6) Describe any assessments undertaken for quality assurance |  |  | ✔ | ✔ |  |  |
| 7) Explain any patient exclusions from analysis |  | ✔ |  | ✔ |  |  |
| 8) Describe how confounding was assessed and/or controlled | ✔ |  |  | ✔ |  |  |
| 9) Explain how missing data were handled |  |  | ✔ | ✔ |  |  |
| 10) Summarize patient response rates and completeness of data collection |  | ✔ |  | ✔ |  |  |
| 11) Clarify follow-up (if any) and incomplete data |  | ✔ |  |  | ✔ |  |

**Note: This checklist evaluates methodological quality across multiple domains relevant to cross-sectional and prevalence studies. Each item is judged as Yes, No, or Unclear based on whether the information is explicitly reported in the study.**

**Supplementary Table 4.** Exploratory subgroup analyses of categorical OR estimates for all-cause mortality

| **Subgroups** | **No. of estimates/cohorts** | **OR**  **(95% CI)** | **Heterogeneity** | | **Effect model** | **P for subgroup difference (Qbetween)** |
| --- | --- | --- | --- | --- | --- | --- |
|  |  |  | **I^2^(%)** | **P-value** |  |  |
| **Study Type** |  |  |  |  |  |  |
| Retrospective cohort | 5 | 3.237(2.373-4.417) | 16.4% | 0.310 | Random | 0.185 |
| Prospective cohort | 2 | 2.864(2.337-3.509) | 82.9% | 0.016 | Random |  |
| **Geographic Region** |  |  |  |  |  |  |
| Asia | 4 | 3.800(2.782-5.190) | 0.0% | 0.431 | Random | 0.042 |
| Western | 3 | 2.788(2.360-3.294) | 54.3% | 0.087 | Random |  |
| **Age** |  |  |  |  |  |  |
| Middle-aged (45-65 years) | 4 | 2.998(2.439-3.684) | 68.4% | 0.023 | Random | 0.562 |
| Elderly (>65 years) | 3 | 2.933(1.988-4.328) | 21.2% | 0.281 | Random |  |
| **Follow up Duration** |  |  |  |  |  |  |
| short-term, 90-day/3-month | 4 | 2.969(2.173-4.057) | 0.0% | 0.411 | Random | 0.659 |
| long-term, >1 year | 3 | 3.043(2.441-3.794) | 78.5% | 0.010 | Random |  |

Note：Subgroup analyses were exploratory because only seven independent estimates were available. P values for subgroup differences were derived from between-subgroup heterogeneity tests. Adjustment status was not examined because most categorical ORs were reconstructed from event counts and were therefore unadjusted.

**Supplementary Table 5.** Exploratory univariable meta-regression for the OR-based analysis of all-cause mortality

| **Moderator** | **Category comparison** | **β** | **SE** | **P-value** | **exp(β), 95% CI** |
| --- | --- | --- | --- | --- | --- |
| Study type | Prospective vs retrospective | -0.134 | 0.175 | 0.443 | 0.874,(0.621-1.232) |
| Geographic region | Western vs Asia | -0.305 | 0.187 | 0.103 | 0.737,(0.511-1.064) |
| Age | Elderly vs middle-aged | -0.011 | 0.215 | 0.960 | 0.989,(0.649-1.509) |
| Clinical setting / follow-up | CV-metabolic/long-term vs acute stroke/short-term | 0.057 | 0.214 | 0.789 | 1.059,(0.696-1.611) |

Note: β values are regression coefficients on the log-OR scale, and exp(β) represents the ratio of ORs between the compared categories. P values were derived from Wald tests in univariable random-effects meta-regression models using restricted maximum likelihood estimation. Multivariable meta-regression was not performed because only seven independent estimates were available.

**Supplementary Table 6.** Harmonization of FIB-4 coding, outcome definitions, and effect estimates

A. Categorical FIB-4 analyses

| **Outcomes** | **Study/cohort** | **Original FIB-4 coding** | **Harmonized contrast** | **Effect estimate (95% CI)** | **Estimate source** |
| --- | --- | --- | --- | --- | --- |
| Overall stroke | Huang et al., 2025 (UK cohort) | Lower cutoff 1.30 or 2.00 by age; upper cutoff 2.67 | Primary: >2.67 vs <=2.67; lower-cutoff sensitivity: >=study-defined lower cutoff vs below | Primary OR 1.925 (1.653-2.242); lower-cutoff OR 1.121 (1.058-1.188) | Event-count ORs; unadjusted |
| Overall stroke | Sun et al., 2024 | Single cutoff 1.30 | FIB-4 >=1.30 vs <1.30 | Adjusted OR 1.570 (1.120-2.260) | Original multivariable OR |
| Overall stroke | Parikh et al., 2019 | Single cutoff 3.25 | FIB-4 >=3.25 vs <3.25 | Adjusted OR 1.870 (1.000-3.500) | Original multivariable OR |
| Ischemic stroke | Song et al., 2023 | Three categories: <1.45, 1.45-3.25, >=3.25 | Primary: >=3.25 vs <3.25; lower-cutoff sensitivity: >=1.45 vs <1.45 | Primary OR 1.843 (0.888-3.825); lower-cutoff OR 1.282 (0.867-1.895) | Reconstructed from event counts; unadjusted |
| Ischemic stroke | Huang et al., 2025 (UK cohort) | Lower cutoff 1.30 or 2.00 by age; upper cutoff 2.67 | Primary: >2.67 vs <=2.67; lower-cutoff sensitivity: >=study-defined lower cutoff vs below | Primary OR 2.045 (1.723-2.428); lower-cutoff OR 0.845 (0.788-0.907) | Event-count ORs; unadjusted |
| Ischemic stroke | Wang et al., 2025 | Single cutoff 2.67 | FIB-4 >2.67 vs <=2.67 | Adjusted HR 1.940 (1.700-2.220) | Original multivariable Cox estimate |
| Ischemic stroke | Kim et al., 2022 | Single cutoff 3.25 | FIB-4 >=3.25 vs <3.25 | Adjusted HR 1.150 (0.780-1.710) | Original multivariable Cox estimate |
| Ischemic stroke | Parikh et al., 2021 | Study-reported categorical cutoff 1.45 | FIB-4 >=1.45 vs <1.45, as reported | Adjusted HR 1.440 (0.490-4.280) | Original multivariable Cox estimate |
| sICH | Gao et al., 2025 | Three categories: <1.30, 1.30-2.67, >2.67 | Primary: >2.67 vs <=2.67; lower-cutoff sensitivity: >=1.30 vs <1.30 | Primary OR 3.070 (1.845-5.105); lower-cutoff OR 1.726 (0.868-3.431) | Reconstructed from event counts; unadjusted |
| sICH | Toh et al., 2023 | Three categories: <1.30, 1.30-2.67, >2.67 | Primary: >2.67 vs <=2.67; lower-cutoff sensitivity: >=1.30 vs <1.30 | Primary OR 2.474 (1.318-4.642); lower-cutoff OR 2.418 (1.186-4.929) | Reconstructed from event counts; unadjusted |
| sICH | Norata et al., 2023 | Three categories: <1.30, 1.30-2.67, >2.67 | Primary: >2.67 vs <=2.67; lower-cutoff sensitivity: >=1.30 vs <1.30 | Primary OR 2.531 (1.034-6.195); lower-cutoff OR 2.304 (1.094-4.852) | Reconstructed from event counts; unadjusted |
| sICH | Çadırci et al., 2025 | Single cutoff 2.67 | FIB-4 >2.67 vs <=2.67 | OR 1.300 (0.502-3.366) | Event-count OR; unadjusted |
| Poor functional outcome | Çadırci et al., 2025 | Single cutoff 2.67 | FIB-4 >2.67 vs <=2.67 | Adjusted OR 2.130 (1.540-4.730) | Original adjusted OR |
| Poor functional outcome | Fandler-Höfler et al., 2021 | Single cutoff 2.67 | FIB-4 >2.67 vs <=2.67 | Adjusted OR 2.150 (1.210-3.830) | Original adjusted OR |
| Poor functional outcome | Gao et al., 2025 | Three categories: <1.30, 1.30-2.67, >2.67 | Primary: >2.67 vs <=2.67; lower-cutoff sensitivity: >=1.30 vs <1.30 | Primary OR 3.545 (2.223-5.651); lower-cutoff OR 2.356 (1.464-3.790) | Reconstructed from event counts; unadjusted |
| Poor functional outcome | Yang et al., 2023 | Three categories: <1.45, 1.45-3.25, >=3.25 | Primary: >=3.25 vs <3.25; lower-cutoff sensitivity: >=1.45 vs <1.45 | Primary OR 2.560 (1.476-4.440); lower-cutoff OR 1.590 (0.829-3.050) | Reconstructed from event counts; unadjusted |
| Poor functional outcome | Toh et al., 2023 | Three categories: <1.30, 1.30-2.67, >2.67 | Primary: >2.67 vs <=2.67; lower-cutoff sensitivity: >=1.30 vs <1.30 | Primary OR 3.185 (2.182-4.650); lower-cutoff OR 2.439 (1.848-3.220) | Reconstructed from event counts; unadjusted |
| Poor functional outcome | Norata et al., 2023 | Three categories: <1.30, 1.30-2.67, >2.67 | Primary: >2.67 vs <=2.67; lower-cutoff sensitivity: >=1.30 vs <1.30 | Primary OR 2.934 (1.302-6.613); lower-cutoff OR 2.222 (1.358-3.636) | Reconstructed from event counts; unadjusted |
| Poor functional outcome | Eto et al., 2024 | Three categories: <1.30, 1.30-2.67, >2.67 | Primary: >2.67 vs <=2.67; lower-cutoff sensitivity: >=1.30 vs <1.30 | Primary OR 2.950 (2.366-3.677); lower-cutoff OR 3.045 (2.197-4.216) | Reconstructed from event counts; unadjusted |
| All-cause mortality | Çadırci et al., 2025 | Single cutoff 2.67 | FIB-4 >2.67 vs <=2.67 | OR 2.115 (0.892-9.641) | Event-count OR; unadjusted |
| All-cause mortality | Fandler-Höfler et al., 2021 | Single cutoff 2.67 | FIB-4 >2.67 vs <=2.67 | Adjusted OR 2.160 (1.160-4.030) | Original adjusted OR |
| All-cause mortality | Yang et al., 2023 | Three categories: <1.45, 1.45-3.25, >=3.25 | Primary: >=3.25 vs <3.25; lower-cutoff sensitivity: >=1.45 vs <1.45 | Primary OR 2.221 (0.841-5.863); lower-cutoff OR 2.274 (0.508-10.188) | Reconstructed from event counts; unadjusted |
| All-cause mortality | Toh et al., 2023 | Three categories: <1.30, 1.30-2.67, >2.67 | Primary: >2.67 vs <=2.67; lower-cutoff sensitivity: >=1.30 vs <1.30 | Primary OR 3.742 (2.480-5.645); lower-cutoff OR 3.605 (2.207-5.887) | Reconstructed from event counts; unadjusted |
| All-cause mortality | Song et al., 2023 | Three categories: <1.45, 1.45-3.25, >=3.25 | Primary: >=3.25 vs <3.25; lower-cutoff sensitivity: >=1.45 vs <1.45 | Primary OR 4.637 (2.677-8.033); lower-cutoff OR 2.924 (1.956-4.371) | Reconstructed from event counts; unadjusted |
| All-cause mortality | Huang et al., 2025 (UK cohort) | Lower cutoff 1.30 or 2.00 by age; upper cutoff 2.67 | Primary: >2.67 vs <=2.67; lower-cutoff sensitivity: >=study-defined lower cutoff vs below | Primary OR 2.605 (2.398-2.829); lower-cutoff OR 1.163 (1.125-1.202) | Event-count ORs; unadjusted |
| All-cause mortality | Huang et al., 2025 (US cohort) | Lower cutoff 1.30 or 2.00 by age; upper cutoff 2.67 | Primary: >2.67 vs <=2.67; lower-cutoff sensitivity: >=study-defined lower cutoff vs below | Primary OR 3.208 (2.768-3.717); lower-cutoff OR 1.535 (1.412-1.668) | Event-count ORs; unadjusted |
| All-cause mortality | Wang et al., 2025 | Single cutoff 2.67 | FIB-4 >2.67 vs <=2.67 | Adjusted HR 2.590 (2.460-2.730) | Original multivariable Cox estimate |
| All-cause mortality | Kim et al., 2022 | Single cutoff 3.25 | FIB-4 >=3.25 vs <3.25 | Adjusted HR 1.420 (1.180-1.710) | Original multivariable Cox estimate |

B. Continuous FIB-4 analyses

| **Outcomes** | **Study** | **Original exposure scale** | **Harmonized scale** | **Effect estimate (95% CI)** | **Adjustment** |
| --- | --- | --- | --- | --- | --- |
| Overall stroke | Xiong et al., 2022 | Adjusted HR per 1-SD increase; FIB-4 SD = 0.9 | Converted to per 1-unit increase using HR_unit = HR_SD^(1/SD) | HR 1.078 (1.022-1.145) | Multivariable adjusted |
| Overall stroke | Caldonazo et al., 2025 | Per 1-unit increase | Per 1-unit increase | HR 1.090 (0.820-1.450) | Multivariable adjusted |
| sICH | Xu et al., 2024 | Per 1-unit increase | Per 1-unit increase | OR 1.306 (1.127-1.512) | Multivariable adjusted |
| sICH | Chen et al., 2024 | Per 1-unit increase | Per 1-unit increase | OR 1.358 (1.184-1.569) | Multivariable adjusted |
| Poor functional outcome | Eto et al., 2024 | Per 1-unit increase | Per 1-unit increase | OR 1.110 (1.030-1.200) | Multivariable adjusted |
| Poor functional outcome | Gao et al., 2025 | Per 1-unit increase | Per 1-unit increase | OR 1.380 (1.110-1.770) | Multivariable adjusted |
| Poor functional outcome | Yang et al., 2023 | Per 1-unit increase | Per 1-unit increase | OR 1.200 (1.060-1.370) | Multivariable adjusted |
| Poor functional outcome | Norata et al., 2023 | Per 1-unit increase | Per 1-unit increase | OR 1.894 (1.160-3.094) | Multivariable adjusted |
| Poor functional outcome | Zhu et al., 2024 | Per 1-unit increase | Per 1-unit increase | OR 1.420 (1.113-1.812) | Multivariable adjusted |
| All-cause mortality | Kim et al., 2022 | Per 1-unit increase | Per 1-unit increase | HR 1.070 (1.050-1.100) | Multivariable adjusted |
| All-cause mortality | Raposeiras-Roubín et al., 2024 | Per 1-unit increase | Per 1-unit increase | HR 1.040 (1.010-1.060) | Multivariable adjusted |
| All-cause mortality | Yang et al., 2023 | Per 1-unit increase | Per 1-unit increase | HR 1.340 (1.080-2.010) | Multivariable adjusted |
| All-cause mortality | Caldonazo et al., 2025 | Per 1-unit increase | Per 1-unit increase | HR 1.190 (1.070-1.320) | Multivariable adjusted |

Note: Categorical ORs reconstructed from group-specific event counts were unadjusted. For studies with two cutoffs, the lower-cutoff comparison combined the intermediate and high groups versus the low group, whereas the upper-cutoff comparison compared the high group versus the low and intermediate groups. The upper-cutoff estimate was used in the primary categorical analysis. ORs and HRs were never pooled together. Incident and prevalent stroke estimates were combined only in the explicitly exploratory any-stroke OR analysis.

Abbreviations: AF, atrial fibrillation; AIS, acute ischemic stroke; ACS, acute coronary syndrome; CI, confidence interval; FIB-4, Fibrosis-4 index; HR, hazard ratio; IVT, intravenous thrombolysis; LVO, large-vessel occlusion; mRS, modified Rankin Scale; MT, mechanical thrombectomy; OR, odds ratio; sICH, symptomatic intracranial hemorrhage; T2DM, type 2 diabetes mellitus.

**Supplementary Table 7.** Eligible effect estimates not included in quantitative synthesis and reasons for non-pooling

| **Study/cohort** | **Outcome** | **FIB-4 coding** | **Effect estimate (95% CI)** | **Reason for non-pooling** |
| --- | --- | --- | --- | --- |
| Wang et al., 2025 (UK Biobank) | Overall stroke | >2.67 vs <=2.67 | Adjusted HR 1.86 (1.66-2.09) | Only one compatible adjusted categorical HR for any stroke remained after outcome-specific classification. It was not combined with ORs or with non-equivalent three-category HR contrasts. |
| Parikh et al., 2025 (UK Biobank) | Hemorrhagic stroke composite | >2.67 vs <=2.67 | Adjusted HR 2.00 (1.60-2.60) | The cohort overlapped with Wang et al. in UK Biobank, and no second independent compatible cohort was available for this exact outcome. Hemorrhagic stroke was not combined with treatment-related sICH. |
| Parikh et al., 2025 (UK Biobank) | Intracerebral hemorrhage | >2.67 vs <=2.67 | Adjusted HR 2.00 (1.50-2.70) | Overlapping UK Biobank source with Wang et al.; after retaining one estimate per independent cohort, fewer than two independent estimates remained for pooling. |
| Parikh et al., 2025 (UK Biobank) | Subarachnoid hemorrhage | >2.67 vs <=2.67 | Adjusted HR 2.20 (1.50-3.50) | Overlapping UK Biobank source with Wang et al.; after retaining one estimate per independent cohort, fewer than two independent estimates remained for pooling. |
| Wang et al., 2025 (UK Biobank) | Intracerebral hemorrhage | >2.67 vs <=2.67 | Adjusted HR 2.14 (1.63-2.81) | Overlapping UK Biobank source with Parikh et al.; no second independent compatible cohort was available. Incident intracerebral hemorrhage was kept separate from post-treatment sICH. |
| Wang et al., 2025 (UK Biobank) | Subarachnoid hemorrhage | >2.67 vs <=2.67 | Adjusted HR 1.90 (1.27-2.84) | Overlapping UK Biobank source with Parikh et al.; no second independent compatible cohort was available. |
| Wang et al., 2025 (UK Biobank) | Stroke-related death | >2.67 vs <=2.67 | Adjusted HR 2.20 (1.73-2.80) | Only one compatible independent estimate was available for stroke-related death; it was not combined with all-cause mortality. |
| Raposeiras-Roubín et al., 2024 | Ischemic stroke | Continuous | HR 1.00 (0.96-1.05) | Only one compatible continuous HR was available for this outcome after strict outcome classification. It was not pooled with categorical HRs or with any-stroke estimates. |
| Xiong et al., 2022 | Overall stroke | Intermediate 1.30-2.67 vs <1.30 | Adjusted HR 1.43 (1.06-1.93) | The published three-category adjusted HR could not be transformed into the prespecified binary contrast >=1.30 versus <1.30 or >2.67 versus <=2.67 without individual participant data or adjusted covariance information. |
| Xiong et al., 2022 | Overall stroke | >2.67 vs <1.30 | Adjusted HR 2.20 (1.43-3.38) | The comparison excluded the intermediate group and therefore did not match the prespecified upper-cutoff binary contrast >2.67 versus <=2.67. An adjusted binary HR could not be reconstructed from published category-specific HRs. |
| Cordero et al., 2023 | In-hospital all-cause mortality | Continuous | Adjusted OR 1.24 (1.04-1.47) | This was the only continuous mortality OR and had an acute in-hospital time horizon. It was not combined with continuous HRs for follow-up mortality. |
| Cordero et al., 2023 | In-hospital all-cause mortality | >2.67 vs <1.30 | Adjusted OR 2.35 (1.045-5.278) | The published contrast excluded the intermediate category and did not match the prespecified binary comparison >2.67 versus <=2.67. Group-specific event data sufficient for reconstruction were not available. |

Note: Estimates from overlapping UK Biobank analyses are displayed separately for transparency but were not treated as independent. No effect estimate was pooled across different effect measures (OR vs HR), incompatible outcome definitions, or non-equivalent FIB-4 contrasts. Cordero et al. also reported no independent association between FIB-4 and postdischarge mortality after adjustment, but no compatible numerical estimate was available for pooling. Abbreviations: CI, confidence interval; FIB-4, Fibrosis-4 index; HR, hazard ratio; OR, odds ratio; sICH, symptomatic intracranial hemorrhage.
